# Spatial Period of Laser-Induced Surface Nanoripples on PET Determines *Escherichia coli* Repellence

**DOI:** 10.3390/nano11113000

**Published:** 2021-11-08

**Authors:** Anja M. Richter, Gerda Buchberger, David Stifter, Jiri Duchoslav, Andreas Hertwig, Jörn Bonse, Johannes Heitz, Karin Schwibbert

**Affiliations:** 1 Bundesanstalt für Materialforschung und -prüfung (BAM), Unter den Eichen 87, 12205 Berlin, Germany; andreas.hertwig@bam.de (A.H.); joern.bonse@bam.de (J.B.); karin.schwibbert@bam.de (K.S.); 2 Institute of Applied Physics, Johannes Kepler University Linz, Altenberger Strasse 69, 4040 Linz, Austria; johannes.heitz@jku.at (J.H.); 3 Center for Surface and Nanoanalytics, Johannes Kepler University Linz, Altenberger Strasse 69, 4040 Linz, Austria; david.stifter@jku.at (D.S.); jiri.duchoslav@jku.at (J.D.)

**Keywords:** laser-induced periodic surface structures (LIPSS), laser processing, polyethylene terephthalate, biofilm formation, cell appendages, F pili, biomimetic

## Abstract

Bacterial adhesion and biofilm formation on surfaces are associated with persistent microbial contamination, biofouling, and the emergence of resistance, thus, calling for new strategies to impede bacterial surface colonization. Using ns-UV laser treatment (wavelength 248 nm and a pulse duration of 20 ns), laser-induced periodic surface structures (LIPSS) featuring different sub-micrometric periods ranging from ~210 to ~610 nm were processed on commercial poly(ethylene terephthalate) (PET) foils. Bacterial adhesion tests revealed that these nanorippled surfaces exhibit a repellence for *E. coli* that decisively depends on the spatial periods of the LIPSS with the strongest reduction (~91%) in cell adhesion observed for LIPSS periods of 214 nm. Although chemical and structural analyses indicated a moderate laser-induced surface oxidation, a significant influence on the bacterial adhesion was ruled out. Scanning electron microscopy and additional biofilm studies using a pili-deficient *E. coli* TG1 strain revealed the role of extracellular appendages in the bacterial repellence observed here.

## 1. Introduction

In their natural habitats, bacteria are usually organized as biofilms: multicellular communities are adhered to surfaces and embedded in a self-produced extracellular matrix consisting of proteins, exopolysaccharides, and extracellular DNA. The matrix not only maintains adherence to the substrate and neighboring cells, but also increases resilience towards antimicrobials and the host immune response [1,2,3,4,5]. Biofilm development is initiated through reversible adhesion of single cells to a provided surface. Initial adhesion, mechano-sensing, and surface locomotion are mediated through nanofiber-like cell appendages such as flagella and pili (also called fimbriae) [6,7,8,9], followed by the production of stabilizing extracellular matrix compounds and maturation into a heterogenous biofilm. 

Within the clinical context, biofilm formation is associated with chronic infections such as e.g., periodontitis, endocarditis or colonization of catheters and medical implants, thus complicating eradication with common antibiotic monotherapy and often resulting in prolonged hospitalization or premature implant failure [10,11,12]. In drinking and wastewater networks and food processing facilities, biofilms facilitate microbial influenced corrosion, biofouling, and deterioration of surfaces as well as product contamination, affiliated with additional economic costs and impaired consumer healthcare [13,14,15,16,17,18].

To prevent persistent microbial colonization, antibacterial surface strategies often target the initial steps of biofilm formation and impede adhesion of single cells before a mature biofilm is being formed [19]. Here, multiple strategies are being pursued involving surface coatings with antimicrobials or metal ions. Those can be released into the medium, promote contact-killing upon bacterial adhesion or induce biofilm dispersal [20,21,22,23]. Some of these surfaces have switchable bacteria-killing and bacteria-releasing properties [24,25]. However, the usage of chemical coating agents should be carefully considered regarding their biocompatibility for eukaryotic cells. In addition, compound-release may lead to attenuated antibacterial-effects and can lead to emerging resistances to antibiotics due to sub-lethal concentrations.

In contrast, intrinsically bacterial-repellent surfaces can be achieved through modified surface topographies [22,26,27,28,29], where laser-fabricated surface structures in the micro- and sub-micrometer range demonstrated both, increased and reduced bacterial retention [30,31,32,33,34,35,36,37]. It was suggested that the specific shape of the bacteria plays a decisive role for their adhesion as well as the depth-to-period aspect ratio of laser-generated micro-structures. While recent publications highlight the anti-biofouling capacities of bioinspired surface structures [38,39] mimicking plant leaves [40], shark and gecko skin [41,42,43] or insect wings [44,45,46], Joel and co-workers demonstrated antiadhesive properties of a nanotextured surface structure bioinspired by cribellate spiders [47]. Here, investigated nanofibers had similar fiber-diameters as bacterial flagella and pili [48], thus, suggesting nano-ripples as a promising strategy to combat nanofiber-mediated bacterial adhesion.

To attain nano-sized topographies on solid surfaces, different laser processing strategies are currently employed. One of the most prominent ones is the self-organized formation of so-called laser-induced periodic surface structures (LIPSS, ripples) featuring spatial periods in the sub-micrometer range [49]. LIPSS formation is a universal phenomenon, observed practically always on solid or liquid surfaces after laser irradiation with polarized light within certain ranges of laser irradiation parameters [50]. In the simplest picture, the formation of LIPSS can be associated with the electromagnetic scattering of the incident laser beam at a microscopically rough surface of a solid material and the resulting optical interference of that radiation with the incident laser light itself, leading to the formation of a corrugated periodic surface relief [51]. Laser pulse durations in the order of some ns, fluences well below the single-pulse ablation threshold, and a large number of laser pulses per irradiated spot have to be applied to induce LIPSS formation on poly(ethylene terephthalate) (PET), poly(styrene), or other polymer surfaces. These LIPSS on polymers depend on the laser wavelength, *λ*, as well as on the angle of incidence, *θ*, of the laser beam [52,53,54,55,56]. For s-polarized laser radiation, the spatial period *Λ* is given by *Λ* = *λ*/(*n*_eff_ - sin*θ*), where *n*_eff_ is the effective refractive index, which lies between the index of air (≈1) and the index of the polymer. The direction of the LIPSS in the case of polymers is usually parallel to the polarization direction and can be classified as a type of low spatial frequency LIPSS (LSFL) [57]. The spatial period *Λ* can be varied by means of different irradiation parameters, but not independently from the structure height (modulation depth), *h*. The aspect ratio of the LIPSS (i.e., *h* divided by *Λ*) is typically between 0.15 and 0.4 here. For more details on the formation of LIPSS, the reader is referred to the following review articles [49,51,57]. A change of surface topography by laser treatment is in most cases also associated with a change of the surface chemistry and surface topography. These alterations (e.g., changes of chemical composition, mechanical properties, hydrophilic/hydrophobic properties, etc.) are certainly different for polymer surfaces, like PET [58,59,60,61], and metal surfaces (steel, titanium, etc.) which have been used for most of the work on bacteria-repellent laser-processed surfaces up to now. For this purpose, we included the surface characterization of the laser-processed PET surfaces by means of spectroscopic methods prominently in our work.

In this work, we generated LIPSS nanostructures with systematically varying spatial periods in the nanometer range (214 to 613 nm) on PET foils and analyzed their bacteria-repellent performance towards *Escherichia coli* (*E. coli*). We demonstrated a strong dependence of bacterial adhesion on structure dimensions and achieved bacterial repellence of almost one order of magnitude. Furthermore, by utilizing genetically different *E. coli* strains, the influence of the LIPSS spatial periods and the specific role of pili on such nanotextured surfaces were addressed, while laser-induced alterations of the surface chemistry could be widely ruled out by spectroscopic methods.

## 2. Materials and Methods

### 2.1. Laser Processing of Poly(Ethylene Terephthalate) (PET) Foils

**Figure 1:**
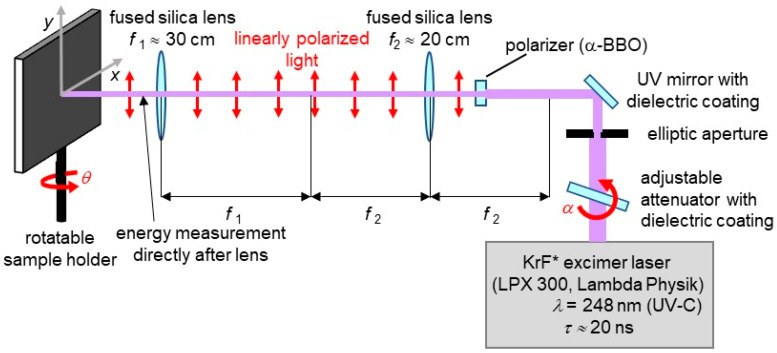
Setup for laser-induced periodic surface structures (LIPSS) fabrication on poly(ethylene terephthalate) (PET) foils by a KrF* excimer laser.

All LIPSS were fabricated on flat, biaxially stretched poly(ethylene terephthalate) (PET) foils with a thickness of 50 µm (Goodfellow Ltd., Bad Nauheim, Germany). In order to form LIPSS on this substrate, a KrF* excimer laser (LPX 300, Lambda Physik, Göttingen, Germany) with a wavelength of *λ* = 248 nm and a pulse duration *τ* of about 20 ns was used (Figure 1). At this wavelength, the optical penetration depth 1/*α* in pristine PET was ~63 nm, with *α*(PET, *λ* = 248 nm) = 1.6 × 10^5^ cm^−1^ being the linear absorption coefficient [49]. The laser pulse repetition frequency *ν* was set to 10 Hz and *N* = 6000 pulses with an average fluence *Φ* of approximately 5.7 to 6.2 mJ/cm^2^ were applied per irradiation spot. The light was linearly polarized with the help of an α-BBO polarizer (Melles Griot, Carlsbad, CA, USA). Two fused silica lenses in a telescope configuration imaged the output of the polarizer onto the samples. A high-power variable attenuator on a rotatable stepper motor with a dielectric coating (magnetron sputtered and with antireflection coating, Laseroptik GmbH, Garbsen, Germany) served for beam energy adjustment. A pyroelectric joulemeter (model ED-500, Gentec from Soliton Laser- und Messtechnik GmbH, Gilching, Germany) was placed after the last lens for beam energy measurements. The samples were glued onto a rotatable sample holder by means of adhesive tape. With the help of this sample holder, the angle of incidence *θ* was increased in steps of 10° from 0° to 60°; this changes the area of the elliptic laser spot according to the formula *A_θ_*_= 0°_⁄cos*θ*, where *A_θ_*_=_ _0__°_ is the area, when the laser hits the sample vertically and when it is not inclined at all. Therefore, the laser pulse energy had to be adjusted accordingly to keep the applied fluence constant. After laser treatment, at least 14 samples were obtained for each value of *θ*, which were then used for morphological, chemical, and microbiological analyses.

### 2.2. Surface Microscopy

#### 2.2.1. Scanning Electron Microscopy (SEM) of PET Foils

All samples have been sputter-coated by gold for SEM imaging (AE1230, EMScope, Ashford, UK; 3 min at a deposition current of 20 mA results in a coating thickness of 8 to 10 nm). After SEM imaging (model REM 1540XB from Zeiss, Oberkochen, Germany), the same samples were used for AFM imaging.

#### 2.2.2. Calculation of Spatial Periods *Λ* from SEM Data

The free software *Gwyddion* (Version 2.55, Czech Metrology Institute, Brno, Czech Republic) served for calculation of the spatial periods *Λ* of the LIPSS. To this end, SEM micrographs taken at one position on the sample with a magnification of 15.14 k-times and with total sizes of 14.83 × 19.77 µm^2^ were first transformed by two-dimensional fast Fourier transforms (2D FFT) using the default conditions (output type “modulus”, windowing type “Hann” and “Subtract mean value beforehand”) and then the profiles along the lines through the resulting peaks were extracted from the transformed images. Lorentzian fits according to the function *f*(*x*) = *y*_0_ + *a*/[*b*^2^ + (*x*−*x*_0_)^2^], where *x*_0_ gives the position of the peak, provided the basis for determination of the distances Δ*k* between the first left and the first right peaks at *k*_l_ and *k*_r_ from the highest center peak, which were then used for calculation of the spatial periods by the formula *Λ* = 2/Δ*k* = 2/(*k*_r_–*k*_l_). From the errors of the peak positions given by *Gwyddion*’s Lorentzian fit function the error bars for *Λ* were calculated by the Gaussian law of propagation of uncertainty. From this we estimated the relevant digits of the values of *Λ*. 

#### 2.2.3. Atomic Force Microscopy (AFM) of PET Foils

Atomic force microscope (AFM) images were obtained with a Digital Instruments CP II setup from Veeco (NY, USA) operated in the non-contact mode (rectangular tip RTESPA-300 from Bruker, Karlsruhe, Germany with nominal/maximal radii of 8/12 nm, minimal/nominal/maximal frequencies of 200/300/400 kHz, minimal/nominal/maximal lengths of 115/125/135 µm, minimal/nominal/maximal widths of 38/40/42 µm and minimal/nominal/maximal spring constants of 20/40/80 N/m); all AFM images were taken moving the tip approximately vertical to the direction of the ripples. Before further usage, AFM data were leveled by mean plane subtraction and the minimum data value was shifted to zero by the respective functions in *Gwyddion*. From high resolution AFM maps from two sample positions showing about two up to fifteen ripples, the height of the LIPSS structures was evaluated by hand using the measurement tool in *Gwyddion* on 10 different ripples from each sample.

### 2.3. X-ray Photoelectron Spectroscopy (XPS)

X-ray photoelectron spectroscopy (XPS) was performed with a ThetaProbe device (Thermofisher, East Grinstead, UK) for a near-surface characterization of the surface composition (at%) and chemical bonding states at the laser-irradiated PET surfaces. To compensate for charging effects, the PET samples were exposed to a stream of low energetic electrons and Ar ions from a dual flood gun during XPS measurements. Photoelectron emission was excited over a nominal spot area with a diameter of approximately 400 µm, using monochromatic focused Al K_α_ X-ray radiation at 1486.6 eV, resulting in an information depth between 5 and 10 nm. For varying the information depth, in selected angular-resolved (AR) measurements the take-off angle of the photoelectrons could be acquired between 20° and 80° with respect to the surface normal. The carbon C1*s* peak at 285.0 eV served as energy reference for charge shift correction of all photoelectron peaks. Survey spectra were recorded in the range between 0 and 1400 eV with an energy step of 1 eV and a pass energy of 200 eV to quantitatively determine surface compositions. Narrow scans with a higher energy resolution (≈0.05 eV steps and 20 eV pass energy) were taken to determine chemical shifts in the C1*s* and O1*s* binding energies, respectively. 

### 2.4. Attenuated Total Reflection Fourier Transform Infrared Spectroscopy (ATR-FTIR)

In order to analyze chemical/structural changes of the PET foils, Fourier-transform infrared spectroscopy (FTIR) was employed before and after the laser processing. The spectra were recorded in attenuated total reflection (ATR) mode of a Hyperion 3000 microscope (Bruker Optik, Ettlingen, Germany) coupled with a Vertex 70 FTIR spectrometer (Bruker Optik, Ettlingen, Germany) using a wavenumber range of 500–3500 cm^−1^ with a resolution of 2 cm^−1^. The ATR microscope objective, equipped with a Ge-crystal tip (reflection angle 45°), increased the surface sensitivity through evanescent field coupling. In this configuration of the ATR mode, the corresponding analytical information depth is well below 1 µm. The ATR-FTIR spectra were acquired with a measurement area of 80 × 80 µm^2^ at various representative positions within the LSFL-covered laser processed surface spots and at the non-irradiated (flat) PET sample as a reference. All ATR-FTIR spectra were measured against a baseline correction in air (without touching any surface with the ATR element). After recording of the spectra, correction algorithms were used in the following order: extended atmospheric compensation removing H_2_O(g) and CO_2_(g) bands, followed by extended ATR correction to compensate for scattering losses and intensity artifacts, followed lastly by a generic baseline correction (concave rubber band method). 

### 2.5. Bacterial Strains and Biofilm Cultivation

For biofilm experiments, *E. coli* TG1 (DSM6056) was chosen, which is a strong biofilm former due to the expression of nanofiber-like F pili (here indicated with the genotype *F^+^*) [32,62]. If not stated otherwise, bacteria were grown in Luria-Bertani (LB) medium at 37 °C on an orbital shaker at 120 rpm or on LB agar plates, respectively. To analyze the effect of pili-dependent attachment and to generate a F-pili deficient strain (*E. coli* TG1 *F^-^*)*, E. coli* TG1 was cured from F plasmid using Acridine Orange (AO) as described previously [63]. A 20 mL LB overnight culture was diluted 1:100 in a total volume of 5 mL LB, supplemented with filter-sterilized AO at concentrations ranging from 1 to 200 µg/mL and incubated overnight. Cell cultures were then diluted, spread onto LB agar plates and incubated again to receive single colonies. Forty colonies were further purified and screened for a phenotype defective in biofilm formation using standard crystal violet assay [64]. To ensure that the biofilm-deficiency was related to the lack of F pili expression, the F plasmid loss was verified using polymerase chain reaction (PCR) and oligonucleotides specific for the *traA*-gene (*traA*-fw 5′- GGTAACTTATGAATGCTGTTTTAAGTG-3′ and *traA*-rev 5′- CAATCCTGGTATCAGTTCTATTTAATC-3′), resulting in a 717 base pair fragment in *F^+^* cells. *traA* encodes for pilin, the pilus subunit protein, and is located on the F plasmid, thus, resulting in PCR-negative results for plasmid-deficient cells.

In general, cell culturing conditions and biofilm cultivation were performed as described by Schwibbert et al. [32] with minor modifications. We inoculated 20 mL of LB medium with *E. coli* TG1 single colonies. After overnight incubation, the culture was 1:100 diluted in fresh, pre-warmed LB and incubated for additional 2 h at 37 °C until cells reached exponential growth phase (at optical density OD_600nm_, approximately 0.3–0.7). Cells were collected by centrifugation (3 min, 5000 g) and diluted to 10^5^ cells/mL in M9 minimal medium [65] supplemented with L-proline (1.7 µM), thiamine (1 mM) and glucose (10 mM). 

PET foils were fixated on glass microscope slides and sterilized with UV light for 15 min. For all experiments, each PET sample contained both, laser-irradiated and non-irradiated areas, to directly compare changes in cell attachment on laser processed surfaces with pristine PET. Samples were placed into six-well multi-dish plates, covered with 5 mL of the prepared bacterial suspension, and transferred into an incubator. After 1 h initial settlement at 28 °C without shaking, biofilm growth was allowed for an additional 21 h under mild shaking conditions (50 rpm) ensuring weak shear forces in the suspension liquid. After a total incubation time of 22 h, OD_600nm_ was documented to monitor general overnight growth of the bacterial population. Non-attached cells were removed by washing with phosphate buffered saline (PBS, pH 7.4), where five times half of the total volume was carefully removed and replaced with fresh PBS. Afterwards, biofilm specimens were further processed for optical light or scanning electron microscopy, respectively.

### 2.6. Optical Light Microscopy and Biofilm Quantification

As the native autofluorescence of the pristine and the laser-structured PET material does not allow epi-fluorescence microscopy, biofilm imaging was performed using optical light microscopy. For quantification of attached cells on PET foils, PBS-washed biofilms were dried, and heat fixated for 30 min at 45 °C. Attached biofilms cells were stained with an aqueous 0.1% Safranin Red solution for 30 min, washed in water to remove excessive staining solution, and finally dried in air. Microscopic observations were performed using a Nikon Eclipse Ni-U (Nikon, Tokyo, Japan) equipped with a CFI Plan Apochromat VC 60 × /1.4 objective. Images were acquired with a Nikon DS-Fi1c camera (Tokyo, Japan) and processed using Nikon’s *NIS Elements* imaging software (Version 4.20). For quantification of biofilm-covered PET surfaces, a total of 25 reflected light micrographs with total sizes of 160 × 213 µm^2^ each were acquired randomly for each condition, i.e., on pristine and laser processed PET areas, and further analyzed using *ImageJ* (Version 1.48v, National Institute of Health, Bethesda, MD, USA) software’s particle count analysis tool. All experiments were performed at least three times for each value of *θ* with different PET samples in each biofilm characterization setup and quantification results for laser processed PET surfaces were compared with results from non-irradiated (pristine) areas of the same PET sample. 

### 2.7. Scanning Electron Microscopy (SEM) of Attached Biofilms

Biofilm fixation was performed as described in [66] with the following modifications. Biofilms on laser-structured PET films were transferred into 1.6% osmium tetroxide in PBS (pH 7.4) and incubated for 45 min at room temperature to allow fixation. After four additional washing steps with PBS, specimens were dehydrated in a graded alcohol series (30%, 50%, 70%, 90%, and 99% ethanol). Critical point drying was performed with liquid carbon dioxide as a transitional fluid (EM CPD300, Leica, Wetzlar, Germany). Biofilm specimens were sputter coated with a 15 nm conducting layer of gold in an EM ACE600 table-top coater (Leica, Wetzlar, Germany) and examined with a Zeiss Supra 40 electron microscope (Zeiss, Jena, Germany) equipped with a field-emission Schottky electron source and operated at an electron accelerating voltage of 5 kV under high vacuum conditions using an In-Lens detector for imaging. A minimum of eight random fields per surface condition (non-irradiated/laser processed) were analyzed.

## 3. Results

### 3.1. Topographic Characterization of Laser Processed PET Foils 

**Figure 2:**
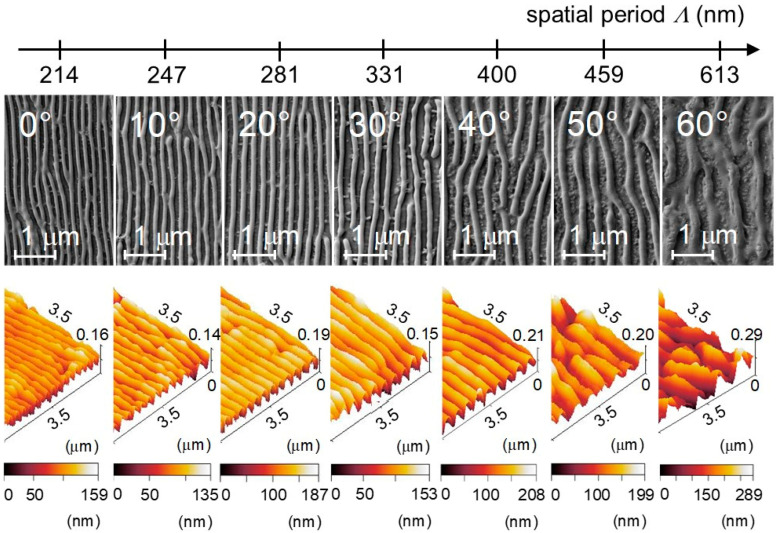
Variation of the spatial period of LIPSS on PET as a function of the angle of incidence *θ* = 0°, 10°, 20°, 30°, 40°, 50°, and 60°of the linearly polarized laser beam at a constant fluence of about *Φ* = 5.7–6.2 mJ/cm^2^, with *N* = 6000 pulses: (**top**) top-view scanning electron microscopy (SEM) images; (**bottom**) atomic force microscopy (AFM) topography images of the corresponding structures.

LIPSS on PET (Goodfellow, 50 µm, biaxially stretched) with different spatial periods *Λ* have been fabricated by the KrF* laser setup with a fluence of about 5.7–6.2 mJ/cm^2^ and 6000 nanosecond laser pulses. Variable spatial periods have been achieved by a change in the angle of incidence *θ* of the linearly polarized light. The structures have been characterized by SEM and non-contact AFM (Figure 2 and Figure S1). From AFM and SEM images the spatial periods *Λ*, the structure heights *h* and several other roughness parameters have been assessed (Table 1 and Table S1).

From the theoretical formula for LIPSS formation on polymers provided in Section 1, the effective refractive index can be calculated by *n*_eff_ = *λ*/*Λ* + sin*θ* to be 1.235 ± 0.053 (±4.3%, seven measurement points) at the applied average fluences of approximately 5.7 to 6.2 mJ/cm^2^. The literature value of *n*_eff_ = 1.32 given in [67] was achieved at a higher fluence of 10.5 mJ/cm^2^, which might explain the discrepancy; a further indication for this is that in LIPSS fabricated at a fluence of approximately 8.5 to 8.8 mJ/cm^2^ the spatial periods calculated with the same method was different, namely 327 nm.

### 3.2. XPS Chemical Characterization of Laser Processed PET Foils

**Figure 3:**
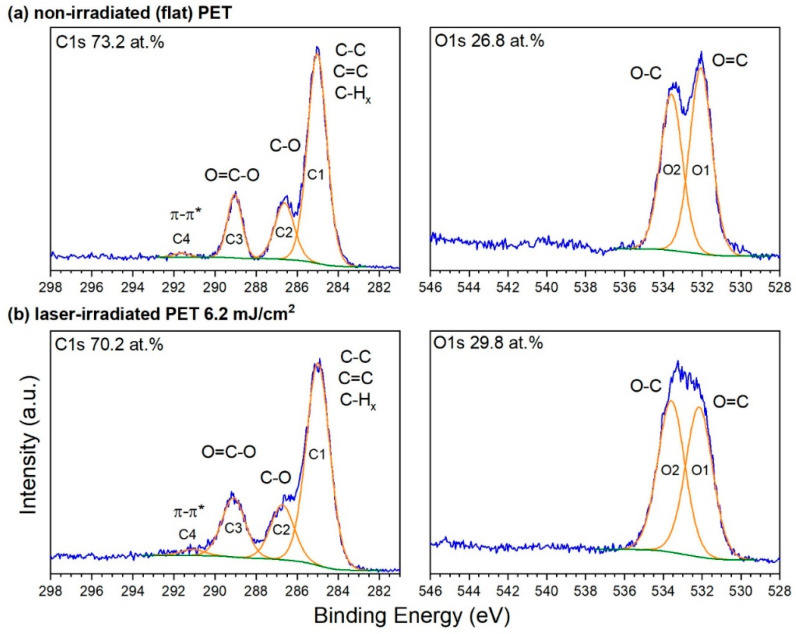
Narrow X-ray photoelectron spectroscopy (XPS) spectra of carbon (C1s) and oxygen (O1s) of non-irradiated (**a**) and ns-laser processed PET samples with low spatial frequency LIPSS (LSFL)-ripples irradiated with a fluence of *Φ* = 5.7–6.2 mJ/cm^2^ at an incidence angle of the laser beam of *θ* = 30° and with *N* = 6000 pulses (**b**). The origin of all peaks is provided in Table S2.

Figure 3 compiles XPS measurements of the carbon C1*s* and oxygen O1*s* atomic core levels that were taken at the surface of non-irradiated PET foil as a reference (a) and at the LSFL-covered surface irradiated at an angle of incidence of *θ* = 30° by *N* = 6000 linearly polarized laser pulses at a fluence of about *Φ* = 5.7–6.2 mJ/cm^2^ (b).

For evaluation, the XPS core level spectra were deconvoluted by fitting Gaussian-Lorentzian peaks (orange lines in Figure 3) after subtracting a Shirley-type background (green lines). The corresponding binding energies of the indicated peaks (labelled C1–C4 and O1, O2) are listed in Table S2 (Supplementary material) as taken from literature [68,69]. 

Some characteristic differences can be observed between the XPS spectra taken in the non-irradiated (Figure 3a) and the laser-irradiated (Figure 3b) surface regions. First, there is a remarkable increase of the C3:C2 ratio of these peak areas in laser irradiated regions to 1.1:1 (Figure 3b). In comparison, the original C3:C2 ratio in the non-irradiated reference sample is found to be only 0.8:1. Second, the C1*s* peaks of the laser irradiated samples exhibit a broadening, which can be explained by the introduction and coexistence of different but spectroscopically similar functionalities, e.g., ester and carboxyl groups. The same is true for the O1*s* spectral region, where also a peak broadening occurs along with a change of the O1:O2 ratio for the irradiated samples from originally 1.2:1 to ~1:1. Third, an observed increase of the oxygen versus carbon content for the laser irradiated samples with respect to the untreated one, taken from the XPS survey spectra and given in Figure 3, is consistent with an increased laser-induced concentration of carboxyl/ester-like groups shown in the narrow scan data. 

Finally, AR-XPS data taken parallel as well as perpendicular to the ripple structure of the laser irradiated samples revealed that there are no significant differences in the chemical composition of the top of ripples compared to their valleys and that there are also no gradients in elemental composition or chemistry within the information depth of XPS, i.e., over the topmost ~10 nm. This is not in accordance with the results from Siegel et al. [70], where a difference between valleys and tops of the ripples was recorded by XPS: however, maybe the reason for this difference is that in our case another fluence was applied which caused differences in chemical surface modification. 

### 3.3. ATR-FTIR Structural Characterization of Laser Processed PET Foils

**Figure 4:**
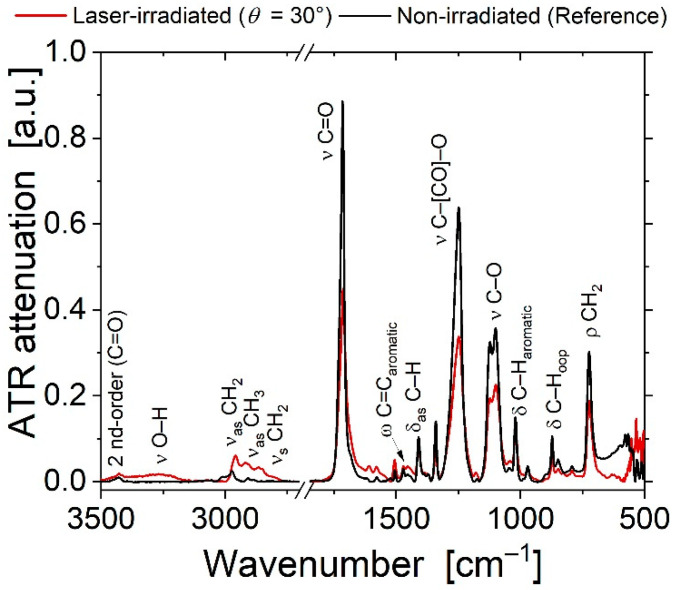
Attenuated total reflection Fourier transform infrared spectroscopy (ATR-FTIR) spectra of non-irradiated PET (black curve) compared to samples processed with LIPSS (type LSFL, red curve, *θ* = 30°, *Φ* = 5.7–6.2 mJ/cm^2^, *N* = 6000 pulses). Note the broken horizontal scale.

Figure 4 shows ATR-FTIR spectra taken at the PET surfaces irradiated at *θ* = 30° (red curve) and at the non-irradiated (flat) PET as a reference (black curve) in two distinct wavenumber ranges, i.e., between 2700 and 3500 cm^−1^ (left part) and between 500 and 1850 cm^−1^ (right part). Several peaks and bands can be observed and are attributed to individual vibrational modes of the probed material, following the assignment of Roberts et al. [71], as compiled in Table S3 (Supplementary material). The symbols ν_s/as_, δ, ρ, and ω denote symmetric or asymmetric radial stretching vibrations (ν_s/as_), latitudinal scissoring (δ) or rocking (ρ) modes, or wagging vibrations (ω), respectively.

Pronounced laser-induced spectral changes are visible and in close agreement with the observations reported by Falkenstein et al. [72] for the irradiation of PET by radiation emitted from a xenon arc lamp, equipped with an IR filter and a UV cutoff at 250 nm. In brief, upon LIPSS processing at *θ* = 30°, a broad band arises between 3130 and 3400 cm^−1^, which can be associated with O–H stretching vibrations either from the –OH or –COOH functional groups [73]. This absorption band can be ascribed to an increased content of hydroxyl or carboxyl groups formed by the UV laser treatment. Their formation is caused by the breakage of the C–O ester group in the PET polymer main chain [72]. Simultaneously, the signal of several different C–H stretching modes increases in the region between 2750 and 3000 cm^−1^ upon laser exposure. Moreover, the signals of strong peaks at ~1716 (–C=O), ~1248 (C–[CO]–O; C–C–O stretch), ~1110 cm^−1^ (–C–O; O–C–C stretch) (all being also characteristic for esters), and the peak at 723 cm^−1^ (–C–H_2_) all decrease along with a characteristic peak broadening. These observations are indicative of the formation of vinyl end groups (–CH=CH_2_) or aldehyde groups (–CHO) [72]. The increase in intensity and complexity within the C–H stretching region hints at chain-breaking reactions not only near the ester function of the PET polymer. It is possible that, additionally, the aliphatic chains or aromatic rings are destroyed to a certain extent. Hence, our ATR-FTIR spectra support a photochemical laser-induced oxidative degradation of PET via photo-dissociation and radical formation, finally leading to the creation of carboxyl, vinyl and aldehyde end groups either through Norrish type I or type II reactions [73,74].

The general spectroscopic trends discussed in Figure 4 remain widely valid also for the irradiations at the other angles of incidence *θ* between 0° and 60° (see Figure S2, Supplementary material). In summary, from the ATR-FTIR measurements similar changes of the near-surface chemistry is found for all laser irradiated spots, which distinguishes them from the non-irradiated PET material. This effect does not differ significantly between the samples and is in line with changes observed by XPS analyses.

### 3.4. Bacterial Attachment on Laser Processed PET

**Figure 5:**
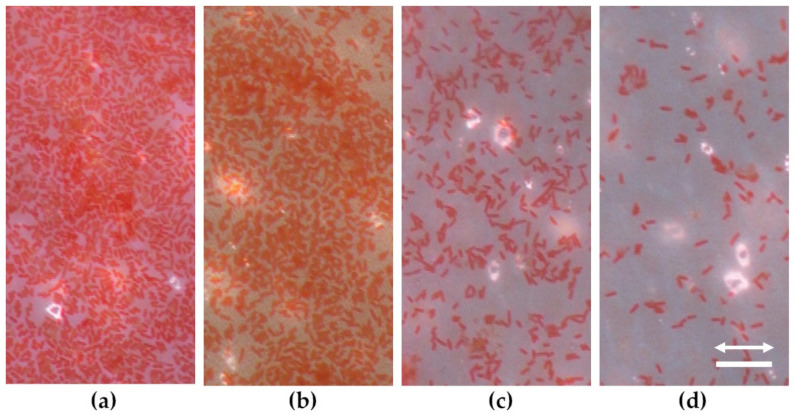
Top-view optical reflected light microscopy of Safranine-stained *E. coli* TG1 on PET foils: (**a**) non-irradiated PET (flat reference); (**b**) laser-irradiated at *θ* = 60°; (**c**) *θ* = 30°; (**d**) *θ* = 0°, resulting in LIPSS periods of 613 nm, 331 nm and 214 nm, respectively. For detailed laser processing parameters see also Figure 2. Scale bar 10 µm. The arrow indicates horizontal LIPSS orientation in (**b**–**d**).

To evaluate the effect of laser-structuring of LIPSS on biofilm formation, *E. coli* TG1 was incubated together with PET foils and allowed to adhere to non-irradiated and laser-structured surfaces. Biofilm cultivation conditions and incubation time were precisely chosen in order to stimulate the formation of a thin biofilm layer of *E. coli* TG1 cells on pristine, non-irradiated PET foils (Figure 5a), which allowed us to monitor and evaluate changes in adherence capacity of the test strain on the laser-irradiated PET in both ways, i.e., with higher coverage rates as well as with lower coverage rates compared to the pristine control surface.

Compared to non-irradiated surfaces, laser-structuring of PET foils resulted in a significantly decreased cell adhesion (Figure 5b–d). LIPSS periods of *Λ* = 613 nm (at *θ* = 60°) only slightly affected biofilm formation and allowed the formation of an almost homogenous monolayer of cells, suggesting bacterial attachment and cell proliferation on the surface. In contrast, *Λ* = 331 nm (*θ* = 30°) LIPSS periods strongly impaired the monolayer formation of bacterial cells. However, on irradiated PET foils with even smaller LIPSS periods of *Λ* = 214 nm (at *θ* = 0°) cell adhesion was strongly reduced with attached single cells and smaller clusters only (Figure 5d). 

**Figure 6:**
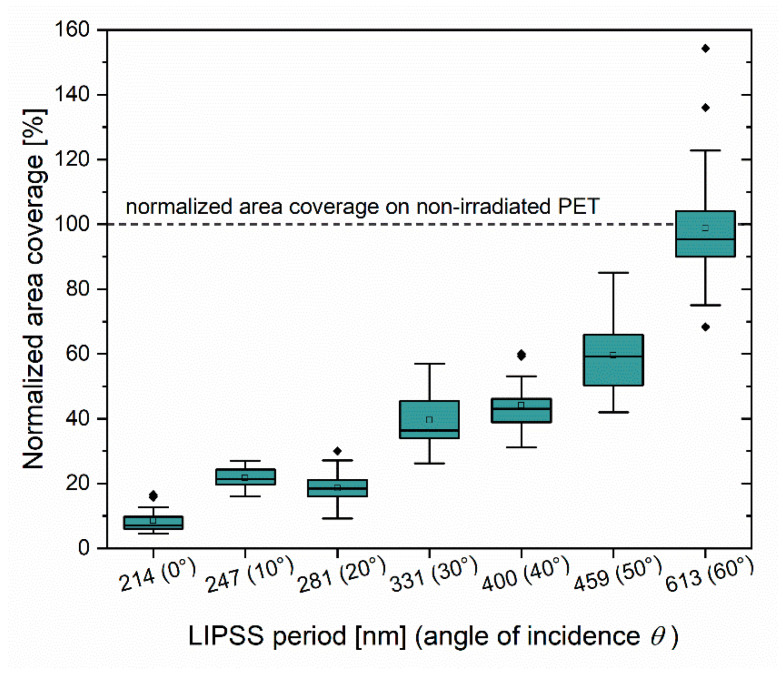
Surface area of laser-irradiated PET covered by *E. coli* TG1 cells normalized against non-irradiated (flat) control surface. Data were obtained through analysis of 25 micrographs of biofilms on laser-irradiated PET and the same number of micrographs of biofilms formed on non-irradiated PET of the same PET foil. The box-whisker plots present 25th and 75th percentiles within the box along with mean (open symbol) and median (continuous line) values, 1.5 interquartile range whiskers, and statistical outliers (closed symbols). All experiments were repeated at least three times for each value of *θ* with different PET samples and the graph displays representative results.

To further quantify the bacterial-repellent effect of different nanostructure periods, optical microscopic images of Safranin-stained bacterial cells were evaluated with the *ImageJ* particle count analysis tool and the results were displayed as area coverage ratios (see Figure 6). Each boxplot shows results from 25 micrographs of biofilms on laser-irradiated PET, which were normalized against data from 25 micrographs of biofilms formed on non-irradiated PET on the identical PET sample. Consistent with optical microscopy images, PET foils structured with LIPSS periods above 600 nm exhibit similar bacterial adhesion rates as non-irradiated PET (<2% absolute change compared to non-irradiated PET), while decreasing LIPSS periods resulted in reduced surface adhesion of *E. coli* TG1. Here, the most considerable reduction in relative cell coverage (−91.4%) was observed for LIPSS periods of 214 nm. In addition, PET foils irradiated at *θ* = 10°, which resulted in 247 nm LIPSS periods showed slightly increased cell adhesion compared to surfaces with 281 nm periods (*θ* = 20°). Detailed AFM analyses indicated alterations of the LIPSS modulation depth (*h*) (comp. Table 1) with the samples irradiated at *θ* = 10°, which resulted in slightly flatter structures (42 ± 15 nm vs. 107 ± 11 nm), thus, suggesting an additional effect of the structure depth on bacterial adhesion. These results suggest that the reduction in cell attachment crucially depends on the LIPSS period (and structure depth) and can be regulated by controlling laser parameters. 

### 3.5. Bacterial Response upon Attachment and Role of Pili

**Figure 7:**
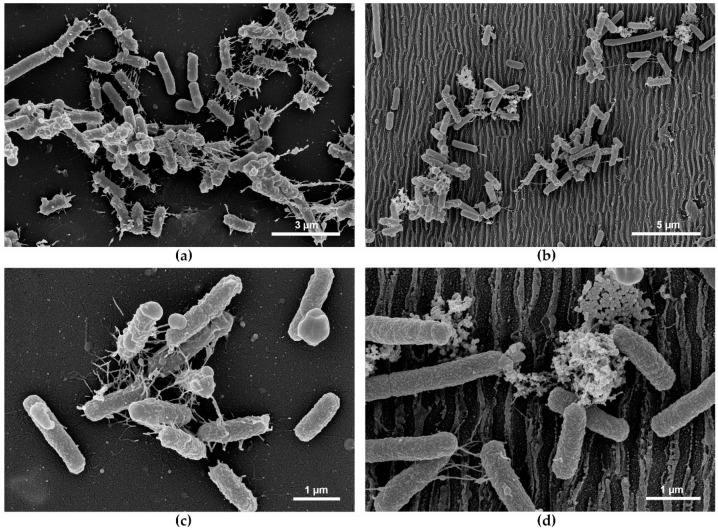
Top-view SEM micrographs of *E. coli* TG1 on (**a**,**c**) non-irradiated PET and (**b**,**d**) laser-structured PET with 214 nm LIPSS spatial period. Arrows in (**c**,**d**) indicate nanofiber-like cell appendages and extracellular matrix, respectively. Note the different scale bars.

To obtain a detailed insight into bacteria–surface interactions, high-resolution scanning electron microscopy imaging of *E. coli* TG1 was performed after biofilm formation on pristine and laser processed PET foils with 214 nm LIPSS periods (*θ* = 0°). SEM micrographs of biofilms on non-structured (flat) PET foils provided in Figure 7a,c reveal *E. coli* TG1 cells with numerous nanofiber-like cell appendages on their surface. The fibers connect individual cells with neighboring ones and also attach the cells to the flat PET surface. In contrast, on LIPSS-covered PET (Figure 7b,d), a granular extracellular material was observed in the vicinity of the bacteria, while the nanofibers established only cell–cell but not cell–surface connections. Lysogenized cells were neither observed on flat, nor on nano-structured PET, suggesting a bacterial-repellent rather than a bactericidal effect of the laser surface structuring. Hence, the SEM results suggested substantial changes in biofilm matrix composition when cells were exposed to nanostructured PET surfaces.

**Figure 8:**
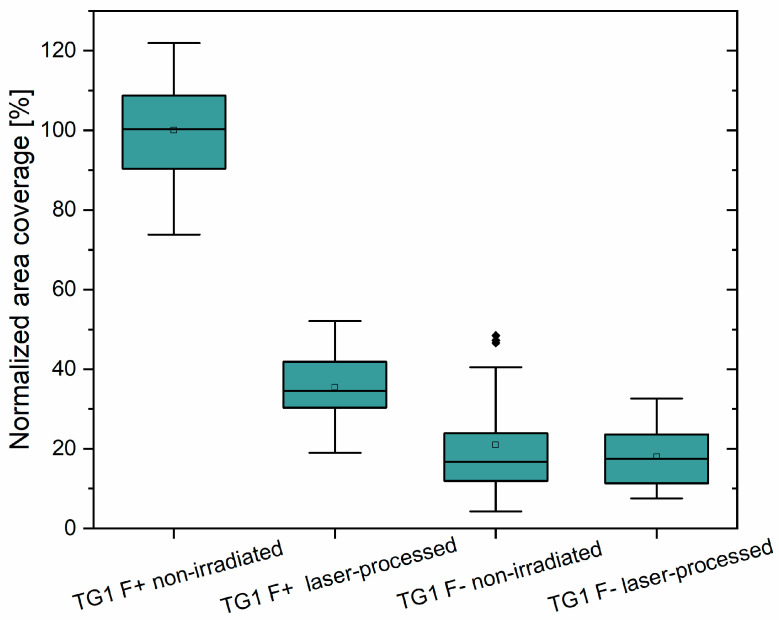
Surface area of pristine and laser-irradiated PET (331 nm, *θ* = 30°) covered by *E. coli* TG1 wild-type (*F^+^*) and plasmid-cured cells (*F^−^*), which do not form pili. Data was normalized against the adhesion results for *E. coli* TG1 wild-type (*F^+^*) on non-irradiated control surface. Box-whisker plots present 25th and 75th percentile within the box along with mean value (open symbol), median value (continuous line), 1.5 interquartile range whiskers and statistical outliers (closed symbols). All experiments were repeated at least three times and graphs display representative results.

*E. coli* TG1 harbors the conjugative F plasmid, which results in the constitutive expression of F pili and renders chromosomal gene expression [75,76]. To further investigate the role of the F plasmid and nanofibrous F pili in surface adherence, we investigated biofilm formation of F plasmid-deficient cells (*F^−^*) on pristine and laser-irradiated PET surfaces. Adhesion experiments on PET foils were performed with LIPSS of 331 nm period. Here, wild-type *E. coli* TG1 (with F plasmid, *F^+^*) showed an average relative surface coverage of 35.4%, which allowed us to monitor and evaluate alterations of plasmid-cured *E. coli* TG1 *F^−^* without pili at higher coverage ratios as well as lower coverage ratios relative to non-irradiated PET. Compared to wild-type *E. coli* TG1, TG1 *F^−^* cells exhibited a decreased adhesion to non-irradiated PET surfaces (21% coverage compared to adhesion of piliated cells (*F^+^*); Figure 8). In addition, bacterial adhesion to laser processed PET foils was reduced in a similar extent (17.9% of wild-type *F^+^* coverage to non-irradiated PET), indicating the relevance of pili for bacterial adhesion to surface-structured as well as pristine PET. However, the coverage rates are far below the levels of the TG1 wild-type incubated on laser-structured PET, which indicates again clearly the role of pili in the adhesion process.

## 4. Discussion

### 4.1. Determination of Surface Physico-Chemical Parameters

The main objective of this study was to evaluate the impact of LIPSS topographies with varying spatial periods on PET surfaces on bacterial adhesion. Surface topography and chemistry are major factors that influence bacterial adherence. Therefore, to assess solely the effects of the surface topography, the surface chemistry has to be known precisely and kept widely constant. To minimize the effect of polysaccharide and protein adsorption, also known as a conditioning layer, which can influence biofilm formation [77], experiments were performed in mineral salt medium with glucose and vitamins as the only organic elements.

Furthermore, XPS chemical characterization and ATR-FTIR structural characterization of the LIPSS-covered laser processed PET foils were carried out. The results revealed that laser processing of the PET surfaces not only results in a change of the surface topography but also in a significant change of the chemical surface composition, both potentially affecting the hydrophilic/hydrophobic surface properties. Nano- or microstructured surfaces often lead to an increase of the water contact angle (compared to flat surfaces) and eventually even to superhydrophobicity (i.e., Cassie-Baxter wetting), like for the leaf of the sacred lotus. This is not the case for laser-structured PET surfaces using the laser parameters of the current study, which lead to significantly increased hydrophilic properties of the nanostructured surface (i.e., via Wenzel wetting or surface oxidation as discussed below). Similar effects of excimer laser treatment on the contact angle and chemical surface composition have been already described in literature [58,59,60]. While the water contact angle (CA) is about CA ~90° for non-irradiated (flat) PET, it is reduced to CA ~60° for PET covered with LIPSS processed at *Φ* = 5.7–6.2 mJ/cm^2^ (data not shown here). For slightly higher fluences (e.g., *Φ* ≈ 8 mJ/cm^2^), where LIPSS are still formed, the water contact angle increases again (to about CA ~75°). This result can be interpreted by the interplay between four processes: (1) material removal by laser sublimation, evaporation, or ablation, (2) oxidation due to the laser irradiation in air, probably after the laser treatment directly at the surface, and (3) photo-induced dissociation of the PET chains, for instance by Norrish type reactions [78], leading eventually to swelling, associated with (4) a pronounced formation of carbonyl (–CO), carboxyl (–COOH), or vinyl (–CH=CH_2_) containing groups. For the lower laser fluence, processes (1) and (2) are dominant, resulting in a surface with an increased content of hydrophilic oxides, while for the higher fluence, processes (3) and (4) gain importance, resulting in again lower hydrophilicity. Obviously, at our fluence levels of around ~6 mJ/cm^2^ laser-induced oxidation plays an important role. These results are corroborated by our XPS and ATR-FTIR measurements on non-irradiated and ns-laser processed PET samples with LIPSS, as previously discussed in detail in Section 3.2 and Section 3.3, respectively. The minor differences between the XPS and the ATR-FTIR data may be mainly attributed to the different information depth of both analytical techniques: While the electron-based XPS technique probes just the topmost few nanometers thick surface layer, the optical method ATR-FTIR acquires its signal over a larger penetration depth up to 1 µm here and, thus, also probes the deeper lying sample regions. Basically, laser processing leads to oxidation of the PET foil surfaces, whereby surface chemistry is substantially the same for all laser-structured samples (*θ* = 0°–60°) as similar fluences (*Φ* = 5.7 to 6.2 mJ/cm^2^) were applied during the laser process. These results are in line with the experiments performed by Nedela et al., who demonstrated via XPS analyses a significant increase of oxygen concentration depending on the fluences applied during the laser processing of polystyrene samples [79]. 

So far, no study has come to our attention that noticed a bacteria repellent effect of oxidized PET surfaces. The opposite seems to be the case as Wilkes and Aristilde propose that an increased hydrophilicity of the polymer facilitates the attachment of bacterial colonies and often the formation of a biofilm requires a plastic polymer to be altered by oxidation [80]. We hypothesize here that a moderate oxidation as observed for the laser processed PET compared to non-irradiated PET samples does not significantly influence bacterial adhesion, neither hampering nor improving it. Moreover, the modest change in hydrophilicity on laser-processed PET does not, in our opinion, influence microbial attachment behavior significantly, since it has been demonstrated that the influence of surface topography dominates surface hydrophobicity when microbial adhesion is considered [33,81]. Consequently, it is reasonable to assume that the observed bacteria repellent effect of laser-structured PET as described in Section 3.4 is primarily based on surface topography and not on surface chemistry or hydrophobicity. 

### 4.2. Bacterial Attachment Depends on the Ripples Spatial Period

Microbial adhesion tests demonstrated that LIPSS structures with periods of approximately 210–460 nm mainly impair *E. coli* TG1 attachment behavior. The degree of bacterial repellence obviously depends on the surface feature density, determined here by the spatial period of the LIPSS. While periods above 600 nm did not cause a bacteria repellent effect, smaller LIPSS periods gave rise to gradually decreased bacterial retention. 

Size and density of surface features strongly influence bacterial adhesion. Numerous studies characterize bacterial attachment on surface features larger than the cell body. Reported results were mainly in accordance with the attachment point theory [82,83], which essentially says that bacteria adhere preferentially to pitches and trenches with most possible attachment points which is particularly the case when features are larger than the cell body [30,31,40,42]. Lutey et al. studied the influence of LIPSS features on steel with periods of basically two sizes, that are smaller or larger than *E. coli* cells. They pointed out that the surface peak density in relation to the size of the bacterial cell body can be considered a simple indicator of the number of available contact points for bacterial cells. For *E. coli* with dimensions of 0.5 × 2 µm^2^, they set the critical surface peak density, where a reduction in *E. coli* surface coverage is expected, to >1/µm^2^ [33]. This is in line with the results of Peter and co-workers. They applied a direct laser interference patterning (DLIP) technique to produce cross wise structures on stainless steel surfaces which efficiently reduced *E. coli* attachment. The authors stressed that a high density of structures on the laser-textured surface (above 1/µm^2^) is fundamental for generating *E. coli* repellent surface topographies [84]. 

However, not only do absolute values of surface feature size and density seem to be important to achieve bacteria repellent surface structures, but also their aspect ratio. Meinshausen et al. generated periodic surface structures on PET foils via the DLIP technique with spatial periods larger than the size of the test strain *S. aureus* (diameter 1 µm, spherical shape) [34]. The authors supposed that the aspect ratio of topographical features is of special importance to accomplish bacteria repellent surfaces and reported minimal bacterial adhesion for an aspect ratio of about 0.02.

The most effective *E. coli* repellent LIPSS structure in our study has an average period of 214 nm. When *E. coli* cells adhere perpendicular to the ridges/grooves of the ripples, the cell body with a dimension of approximately 1–1.5 µm in length under our test conditions, covers about 4–5 ridges (see Figure 7). These results are in line with the studies mentioned above as the peak density value of 4–5/µm exceeds the proposed critical value for *E. coli*. Upon adhesion parallel to the ripples *E. coli* cells with a width of approximately 0.5 µm can cover a single groove. No preferred orientation of bacterial cells was observed which implies that the cell body, despite its cylindrical shape, does not fit in between adjacent ripples. Although the aspect ratio of our most effective structure is roughly 0.3 and, according to Meinshausen et al. [34], not best to achieve *E. coli* repellent surfaces, we demonstrate here a significant reduction of bacterial coverage (~91%). This particular difference may arise from the differences in the absolute values of the spatial LIPSS periods ranging between ~200 nm and ~600 nm used here and above 700 nm or 1.5 µm for the DLIP processed surfaces on titanium or PET, respectively, in Meinshausen et al. 

### 4.3. LIPSS Mainly Affect the Nanofiber-Mediated Adhesion

When considering the density of features as a tool to design surfaces with low bacterial retention capacity, density is usually meant in relation to bacterial size, implicating that bacteria act as small particles. However, this is actually not the case as it disregards biological features such as appendages and adhesins on the cell surface, membrane rigidity and turgor, alternate gene expression due to membrane stress or surface sensing. In our study, all LIPSS periods under investigation were below the size of the test strain *E. coli* TG1 and, therefore, could be predicted to exhibit similar bacterial repellence. However, the extent of bacterial retention differed significantly, which might be explained by the fact that the first contact with a surface is usually mediated via cell appendages, such as flagella or pili and not by the cell body itself. 

Friedlander et al. demonstrated that flagellated *E. coli* achieved better adhesion to structured surfaces with 440-nm trenches, thus smaller than the cell body, compared to flat surfaces [6]. Flagella were able to access the trenches, consequently flagellated wild-type cells experienced an increase in available surface area on structured substrates (compared with flat ones) with excellent attachment possibilities, whereas the non-flagellated mutant cells experienced a decrease. The test strain employed in our study harbors a de-repressed F-plasmid, which leads to an overexpression of nanofiber-like F-pili. F-pili are strong cell–cell as well as cell–surface adherence factors, with whom the bacterial cell actively explores its surrounding and which predominate the effect of all other adhesins including flagella [62,85,86]. The experimental setup in M9 minimal medium was expected to reduce expression of flagella [87]. Therefore, first surface contacts and subsequent attachment of *E. coli* TG1 cells can mainly be attributed to the occurrence of F-pili. Under our test conditions, F-pili seemed to find abundant attachment points on flat surfaces and on ripples with periods above 600 nm, but not on ripples with spatial periods of ~210 to ~460 nm (see Figure 6). Moreover, as visualized by scanning electron microscopy (Figure 7) bacteria exposed to repellent nanostructured PET with a spatial period of 214 nm did not establish stable nanofiber-mediated cell–surface contacts. By contrast, on flat surfaces cell–surface contacts are abundant. These findings are in accordance with previous studies on cribellate spider silk nanofiber adhesion on laser structured PET foils. Joel et al. demonstrated that PET foils structured with LIPSS with spatial periods of approximately 320–340 nm reduced the adhesion forces of nanofibers with a comparable diameter of F pili (10–30 nm for the spider silk [47] vs. ~8–9 nm for the pili [48], respectively).

Results obtained with wild-type *E. coli* TG1 carrying F-pili compared to plasmid-cured *E. coli* TG1 lacking F-pili clearly demonstrate that the extent in adhesion reduction is far more pronounced when LIPSS act on piliated cells. We assume that for *E. coli* TG1 repellence, LIPSS trenches have to be sufficiently narrow and deep, that the limited flexibility of cell appendages (here: F-pili) limits the bacterial access to the surface. In contrast, if surface appendages are able to access trenches, cells may experience an increase in available surface area, thus leading to enhanced bacterial attachment. Whether our result of more than 90% reduction can be improved by further reduced LIPSS periods remains to be investigated. We speculate here that if the period falls below a critical value, bacterial adhesion will gain and resemble adhesion to flat unstructured surfaces.

### 4.4. Extrapolymeric Substances Help to Augment Bacterial Adhesion on Otherwise Unfavorable Surfaces

The SEM micrographs shown in Figure 7 reveal that upon adhesion of *E. coli* TG1 on structured surfaces, granular matrix components are produced that have not been observed on flat surfaces. The studies carried out by Prigent-Combaret et al. indicate that this matrix component could be colanic acid, a viscous capsular exopolysaccharide produced by numerous Enterobacteriaceae [88]. 

Surface topographies that are unfavorable for bacterial adhesion might induce stress to the bacterial cells and lead to enhanced production of matrix components. This is supported by results reported by Mitik-Dineava et al. where a change in nanometer scale roughness resulted in increased production of matrix components in *Pseudomonas issachenkonii* [89]. May and Okabe noticed a F-plasmid mediated upregulation of biofilm matrix components such as colanic acid [76]. Therefore, F plasmid deficient *E. coli* TG1 are not only lacking F-pili but may also show a decreased matrix expression, which additionally hampers adhesion capabilities and might partially explain the poor coverage rate of this strain (Figure 8). Our SEM results suggest substantial changes in biofilm matrix composition when cells were exposed to LIPSS-covered PET surfaces with the result that here, bacteria did not establish stable nanofiber-mediated cell–surface contacts. However, these changes did not compensate for nanofiber-repellent surface characteristics. 

## 5. Conclusions

Many bacteria are equipped with nanofiber-like appendages (e.g., pili and flagella) that mediate the first contact with a surface and act as strong adhesins. However, the outstanding role of cell appendages in surface adhesion has so far not sufficiently been taken into account when bacteria repellent surfaces are in focus. Using laser processing as a tool for surface nano-structuring, adhesion of bacterial cells—and therefore the initial steps of biofilm formation—was strongly impeded. Systematic analysis of a broad range of nanostructures clearly demonstrates the dependence of bacterial adhesion on spatial periods. In addition, our results point out that the efficiency of bacteria-repellent surfaces can be significantly improved when surface structures impair nanofiber-mediated adhesion. To further improve the design of bacteria-repulsive surfaces, structures with the fewest possible attachment points for bacterial nanofibers like flagella and pili must be considered.

## Figures and Tables

**Table 1:** Spatial period *Λ*, height of the ripples *h* ± standard deviations for LIPSS on PET formed with various angles of incidence *θ* of the laser beam (*Φ* = 5.7–6.2 mJ/cm^2^, *N* = 6000 pulses).

Angle of Incidence *θ* [°]	0°	10°	20°	30°	40°	50°	60°
Spatial period *Λ* [nm]	214	247	281	331	400	459	613
height *h* [nm]	64 ± 8	42 ± 15	107 ± 11	84 ± 6	111 ± 18	72 ± 12	117 ± 52

## Data Availability

The data presented in this study are available on request from the corresponding author.

## Supplementary Materials

The following are available online at https://www.mdpi.com/article/10.3390/nano11113000/s1, Figure S1: SEM and AFM images of untreated (flat) PET. Figure S2: ATR-FTIR spectra, Table S1: Roughness parameters, Table S2: XPS peaks, Table S3: FTIR peaks.
