# Supplementary material for: Spatial Period of Laser-Induced Surface Nanoripples on PET Determines Escherichia coli Repellence
Source: Nanomaterials (Basel). 2021 Nov 8;11(11):3000. doi: 10.3390/nano11113000 (PMC8624992; doi:10.3390/nano11113000)
Supplement: Supplementary file 1 [file nanomaterials-11-03000-s001.zip › nanomaterials-1440150-supplementary.pdf]

nanomaterials

Supplementary Materials of

Spatial Period of Laser-Induced Surface Nanoripples on PET Determines *Escherichia coli* Repellence

Anja M. Richter [1,*], Gerda Buchberger [2,*] David Stifter [3], Jiri Duchoslav [3], Andreas Hertwig [1], Jörn Bonse [1], Johannes Heitz [2] and Karin Schwibbert [1]

[1] Bundesanstalt für Materialforschung und -prüfung (BAM), Unter den Eichen 87, 12205 Berlin, Germany; andreas.hertwig@bam.de (A.H.); joern.bonse@bam.de (J.B.); karin.schwibbert@bam.de (K.S.)
[2] Institute of Applied Physics, Johannes Kepler University Linz, Altenberger Strasse 69, 4040 Linz, Austria; johannes.heitz@jku.at (J.H.)
[3] Center for Surface and Nanoanalytics, Johannes Kepler University Linz, Altenberger Strasse 69, 4040 Linz, Austria; david.stifter@jku.at (D.S); jiri.duchoslav@jku.at (J.D.)
[*] Correspondence: anja.richter@bam.de (A.M.R.); gerda.buchberger@jku.at (G.B.) (these authors contributed equally to this article).

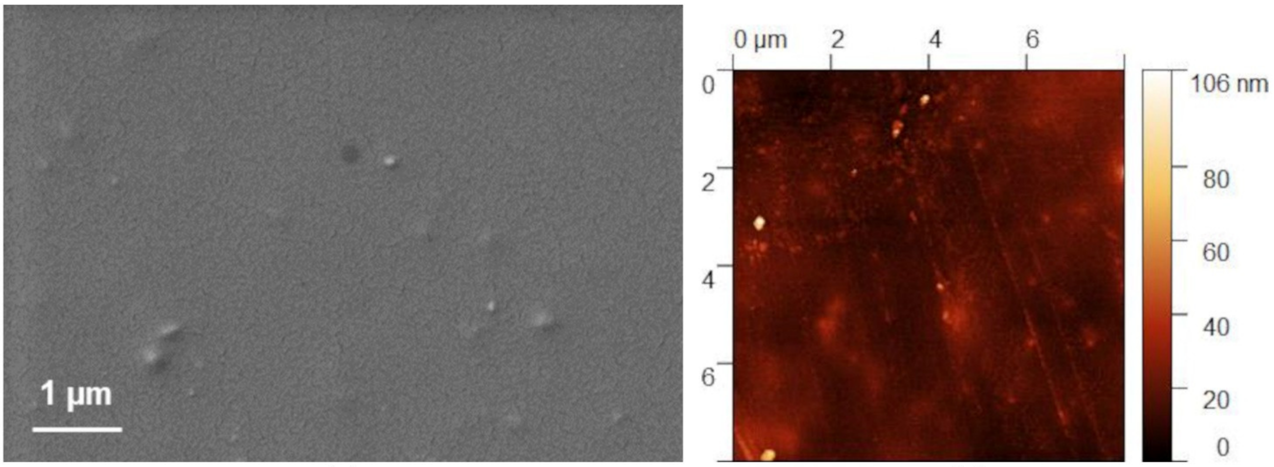

(a) (b)

Figure S1. Top-view SEM (**a**) and AFM topography (**b**) images of non-irradiated (flat) PET serving as a reference sample.

Calculation of roughness parameters from AFM data

The roughness average R_a, the root mean square roughness R_q as well as the average maximum height of the roughness $R_{z\,ISO}$ were calculated by means of the free software *Gwyddion* from eight profile lines from high resolution AFM images with sizes of 1.5 × 1.5 µm² and 3.5 × 3.5 µm² taken on at least two sample positions. To this end, the "Calculate roughness" tool was applied, where the cut-off frequency can be manually adjusted so that surface waviness and surface roughness can be separated. The root mean square roughness R_q is the average of the measured height deviations from the mean line. The roughness average R_a represents the average deviations of the ripples from the center plane of the laser processed samples; as the generated nanoripples are not perfectly symmetric, this value is not directly related to half of the ripple height, wherefor the heights of the ripples were also measured separately by hand. From high resolution AFM maps from two sample positions showing about two up to fifteen ripples, the height of the LIPSS structures was evaluated by hand using the measurement tool in *Gwyddion* on 10 different ripples from each sample. Results are given in Table S1 and errors are the standard deviations of these measurements.

Table S1. Roughness parameters ± standard deviations for LIPSS on PET formed with various angles of incidence θ of the laser beam (Φ = 5.7 - 6.2 mJ/cm^2, N = 6000 pulses).

Angle of incidence θ [°]	0°	10°	20°	30°	40°	50°	60°
Roughness average R_a [nm]	18 ± 1	13 ± 3	31 ± 3	23 ± 2	33 ± 2	23 ± 4	38 ± 5
Root mean square roughness R_q [nm]	22 ± 1	15 ± 4	36 ± 3	27 ± 2	37 ± 3	28 ± 5	46 ± 6
Average maximum height of the roughness $R_{z\,ISO}$ [nm]	71 ± 4	48 ± 14	110 ± 11	94 ± 5	118 ± 8	85 ± 8	135 ± 16

Table S2. Set of peak energies of carbon (C) and oxygen (O) used for the deconvolution of the XPS spectra along with their assignment and chemical origin [1, 2] (see Ref. [68, 69] in the main text).

**Peak label / Element transition**	**Peak energy** [eV]	**Bond / Origin**
C1 / C 1*s*	285.0	C=C, C-C, C-H_x / aromatic carbon or hydrocarbon
C2 / C 1*s*	286.6	C-O, C=O, C-OH / carbon bearing a single oxygen
C3 / C 1*s*	289.1	COOH, COO / carbon in carboxyl group or ester
C4 / C 1*s*	291.5	π-π* / aromatic shake-up
O1 / O 1*s*	532.1	O=C / π-bonded oxygen
O2 / O 1*s*	533.6	O-C / σ-bonded oxygen

Table S3. FTIR peaks along with their assignment and structural origin [3, 4] (see Ref. [71, 73] in the main text).

**Peak/band position [cm^{-1}]**	**Vibrational mode**	**Bond / Origin**
723	ρ C-H_2	CH_2 rocking mode
872	δ C-H_{oop}	C-H scissoring vibrations
1020	δ C-$H_{aromatic}$	C-H scissoring vibrations of aromatic carbon

1110	ν C-O	-C-O; O-C-C stretching vibrations
1248	ν C-[CO]-O	C-[CO]-O; C-C-O stretching vibrations
1410	δ_{as} C-H	CH asymmetric scissoring vibrations
1456	ω $C{=}C_{aromatic}$	C=C wagging vibrations of aromatic carbon
1716	C=O	C=O vibrations
2860	ν_s CH_2	CH_2 symmetric stretching vibrations
2915	ν_{as} CH_3	CH_3 asymmetric stretching vibrations
2960	ν_{as} CH_2	CH_2 asymmetric stretching vibrations
3130 - 3400	ν O-H	OH-stretching vibrations

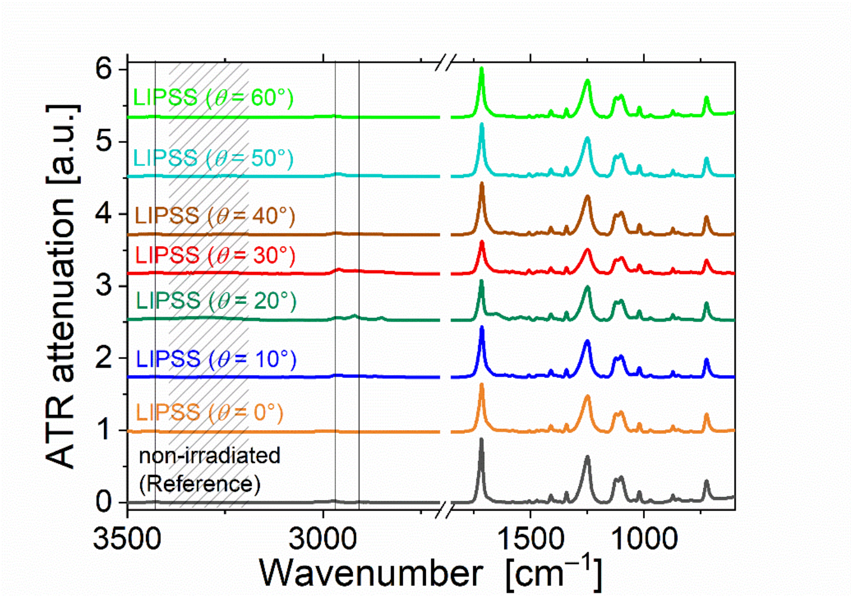

Figure S2. ATR-FTIR spectra of non-irradiated PET (black curve) compared to samples processed with LIPSS (type LSFL, Φ = 6.2 mJ/cm^2, N = 6000 pulses) at different angles of incidence θ = 0°, 10°, 20°, 30°, 40°, 50°, and 60°. Note the broken horizontal scale. The spectra are shifted vertically to facilitate their intercomparison.

References

1. Briggs, D. *Surface Analysis of Polymers by XPS and Static SIMS*. Cambridge University Press, New York, **1998**.
2. Beamson, G.; Briggs, D. "High Resolution XPS of Organic Polymers." In *The Scienta ESCA 300 Database*. Chichester: John Wiley & Sons, **1992**.
3. Roberts, C.; Edwards, S.; Vague, M.; Leon-Zayas, R.; Scheffer, H.; Chan, G.; Swartz, N. A.; Mellies, J. L. Environmental Consortium Containing *Pseudomonas* and *Bacillus* Species Synergistically Degrades Polyethylene Terephthalate Plastic. *Msphere* **2020**, *5*, e01151-01120. https://doi.org/10.1128/mSphere.01151-20.
4. Day, M.; Wiles, D. M. Photochemical Degradation of Poly(ethylene Terephthalate). 2. Effect of Wavelength and Environment on Decomposition Process. *J. Appl. Polym. Sci.* **1972**, *16*, 191-202. https://doi.org/10.1002/app.1972.070160117.
